# AIDS Vaccine Development: the Past, the Present, and the Future

**DOI:** 10.4110/in.2009.9.1.1

**Published:** 2009-02-28

**Authors:** Soshin Ahn, Youngchul Sung

**Affiliations:** Cellular Immunology Laboratory, Division of Molecular and Life Sciences, Pohang University of Science and Technology, Pohang, Korea.

**Keywords:** AIDS, preventive vaccine

AIDS was first reported in 1981 by Gottlieb MS et al at USC (University of California) Medical Center: Healthy homosexual men with pneumocystis carinii pneumonia and mucosal candidiasis had deficiency in CD4 T lymphocyte, a critical immune defense cell (1). In 1983, Dr. F. Barré-Sinoussi and L. Montagnier isolated a new human T-lymphotropic retrovirus, later named HIV-1(human immunodeficiency virus type 1) which turned out to be one of the causes of AIDS (2). Through their discovery, they received the 2008 Nobel Prize in Physiology or Medicine award.

Discovery of the causative agent of AIDS had been the driving force to investigate its genomic structure, function, and replication mechanism. Most importantly, the development of a diagnostic kit contributed to the knowledge of AIDS as an infectious disease especially in the route of transmission. In addition, the development of antiretroviral drugs reduced the mortality of some HIV-infected individuals. However, high price and side effects of the current therapeutic drugs have not been beneficial for most AIDS patients. Thus, it has been generally accepted that the development of a low price and effective prophylactic AIDS vaccine is the only answer to stop the global epidemic. Despite tremendous efforts over the past 25 years, there is no promising candidate for an HIV vaccine. No one has even been able to design and develop a model leading to an effective vaccine. This review will introduce four major milestones in AIDS vaccine development to date which resulted in major social, economic and scientific impacts (Fig. 1).

In 1989, the hopes for a HIV vaccine started from a highly effective formalin-inactivated whole SIV vaccine which was known to confer protection in macaques with AIDS (3). However, three years later, it was found that the protective effect was mediated by antigens (such as HLA and β2m) from the human cells used to grow the viral strain (4).

In 1992, Dr. Ronald Desrosiers reported that a live attenuated SIV vaccine with a deletion in the nef gene can be efficient in treating AIDS of macaques (5). Unfortunately, three years later, Dr. Ruth Ruprecht found that this live attenuated vaccine had a safety issue in which the vaccine itself caused AIDS in neonatal macaques. For this reason, research into the development of a live attenuated vaccine has been no longer carried out (6).

In 1990, Genentech reported that their recombinant glycoprotein gp120 subunit vaccine could induce protection in chimpanzees with HIV-1 (7). In phase I and II trial, they confirmed the safety and immunogenicity. However, when this vaccine was tested on 5,000 volunteers to test efficacy, vaccination failed to show reduction in HIV and thus was not able to become commercialized (8).

Finally, Merck Co. reported that replication-incompetent adenoviral vaccine could elicit effective anti-viral T cell immune response against SHIV (pathogenic HIV-1 and SIV hybrid virus) in 2002 (9). Based on this positive result in monkeys, they entered clinical trial to evaluate the efficacy with almost 10,000 HIV-1 negative healthy volunteers. Disappointedly, in November 2007, they announced negative results on the interim report with 3,000 volunteers. This vaccine was ineffective in lowering plasma viremia postinfection and increased the risk of acquiring HIV-1 infection; therefore, further study on the vaccine was not pursued (10).

There were five major vaccines introduced as possible treatments for AIDS: killed vaccine, live-attenuated vaccine, subunit vaccine, vectored vaccine, and DNA vaccine (Table I). Among them, DNA vaccine is the most promising AIDS vaccine since it was the only one that could provide a safe and protective immunity against HIV. Despite the safety concerns of the live attenuated vaccine, it is currently the only vaccine that is capable of inducing a protective immunity. Hence, the vaccine that has similar qualities and induces a strong response of the immune system similar to that of the live attenuated vaccine is the most probable to become a successful AIDS vaccine. In this regard, the fact that the DNA vaccine with electroporation (EP) does not have a vectored immunity makes it an excellent candidate because it can induce antibodies and a T cell response by continuous injections which are as powerful as live the attenuated vaccine. Therefore, there are high expectations for DNA vaccine with EP to develop successful AIDS vaccines commercially available in the near future.

In order to develop an effective DNA vaccine with EP, the vaccine candidate should be evaluated thoroughly in terms of protective immunity in a small number of volunteers before entering large-scale phase IIb~III efficacy trials. More importantly, even before considering any clinical trials in humans, the efficacy test should be evaluated in the appropriate SIVmac-rhesus macaque challenge model that closely resembles the human case.

## Figures and Tables

**Figure 1:**
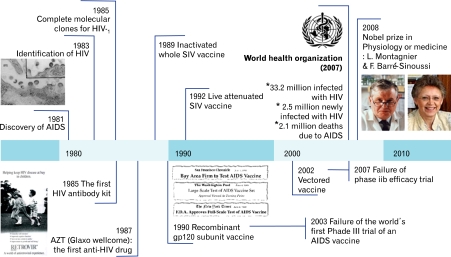
Time lines for HIV/AIDS pandemic and the development for AIDS vaccines.

**Table I:**
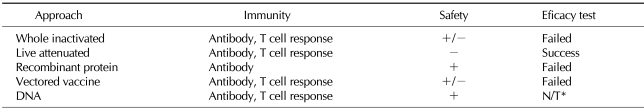
The Potential of the five major AIDS vaccine candidates

^*^N/T: Not tested, +: Safe, +/-: Mild concerns, -: Serious concerns
